# Influence of a Sedentary Behavior Intervention on Mood, Sleep, and Quality of Life Outcomes During Pregnancy: The SPRING Study

**DOI:** 10.1089/whr.2024.0176

**Published:** 2025-03-25

**Authors:** Andrea C. Kozai, Katrina L. Wilhite, Christopher E. Kline, Kelliann K. Davis, Alisse Hauspurg, Janet M. Catov, Bethany Barone Gibbs

**Affiliations:** ^1^ Department of Epidemiology, University of Pittsburgh, Pittsburgh, Pennsylvania, USA.; ^2^ Department of Epidemiology & Biostatistics, West Virginia University, Morgantown, West Virginia, USA.; ^3^ Department of Health and Human Development, University of Pittsburgh, Pittsburgh, Pennsylvania, USA.; ^4^ Department of Obstetrics, Gynecology & Reproductive Sciences, University of Pittsburgh, Pittsburgh, Pennsylvania, USA.; ^5^ Women & Infants Hospital of Rhode Island/Aplert Medical School, Brown University, Providence, Rhode Island, USA.; ^6^ Departments of Obstetrics, Gynecology & Reproductive Sciences, Epidemiology, and Clinical and Translational Science, University of Pittsburgh, Pittsburgh, Pennsylvania, USA.

**Keywords:** perinatal mood, behavior change, accelerometry, maternal health, pregnancy health

## Abstract

**Background:**

Psychological symptoms and sleep disturbance are common during pregnancy. Observational data suggest that being physically active during pregnancy is related to better mood and sleep, but whether sedentary behavior reduction interventions provide similar benefits is untested. We aimed to determine whether reducing sedentary behavior across pregnancy improved psychological and sleep parameters.

**Methods:**

Pregnant participants (*n* = 51) were allocated 2:1 to a sedentary behavior reduction intervention or control in their first trimester. Depressive symptoms, perceived stress, mood disturbance, nausea/vomiting quality of life, and sleep parameters were assessed with validated questionnaires in each trimester. Linear mixed effects regression examined differences between groups across pregnancy. Spearman correlations tested whether changes in sedentary time and physical activity were associated with changes in psychological and sleep outcomes without regard to group.

**Results:**

Despite significant reductions in sedentary behavior (−0.84 hour/day), the intervention had no effect on psychological health outcomes. Further, intervention participants demonstrated significant worsening of sleep efficiency factor scores compared with control (*p* = 0.038). Small but significant correlations were found between changes in sedentary time and nausea/vomiting quality of life, and between changes in physical activity and nausea/vomiting quality of life, sleep duration, and sleep efficiency.

**Conclusions:**

Reducing sedentary behavior during pregnancy did not improve psychological symptoms and may worsen sleep efficiency. Recommendations for future sedentary behavior reduction research in pregnancy include a larger sample with poorer psychological health and sleep at baseline, targeting reductions in mentally passive sedentary behavior, and including device-based sleep assessments.

## Introduction

Pregnancy is a dynamic period, during which many individuals experience worsening of psychological and sleep outcomes. Between 10% and 20% of pregnant individuals develop depression during pregnancy, which can continue postpartum.^1^ Also, many pregnant individuals report being mildly to moderately stressed during pregnancy,^2,3^ and mood instability tends to be higher in pregnant individuals compared with their nonpregnant counterparts.^4^ Additionally, sleep duration and quality are often poor during pregnancy.^5^ Exploring strategies to improve mood and sleep during pregnancy is a priority, as suboptimal mood and sleep are associated with an increased incidence of adverse pregnancy outcomes such as preeclampsia.^1,6–8^ Further, if these issues persist postpartum, there is an increased risk of poor maternal outcomes, such as suicide, and behavioral, cognitive, and health problems in the child.^1,2,4^

Physical activity has been shown to improve many psychological^9,10^ and sleep^11^ outcomes during pregnancy, with the American College of Obstetrics and Gynecology recommending at least 150 minutes of moderate-intensity physical activity per week.^12^ However, fewer than 40% of pregnant people in the United States are estimated to meet this recommendation.^13^ This may be a consequence of barriers pregnant individuals face to being physically active, such as increased fatigue, pregnancy-related discomforts, and lack of time.^14^ The limited number of pregnant individuals meeting physical activity recommendations, coupled with the increased barriers to physical activity, suggests that exploring new strategies to improve psychological and sleep outcomes during pregnancy is necessary.

Sedentary behavior, defined as time spent seated or lying down with low energy expenditure, is distinct from physical activity. This can be understood by the fact that it is possible to be both highly active and highly sedentary, for example, by exercising daily after sitting all day at work. Furthermore, sedentary behavior appears to confer risk for poor psychological and pregnancy outcomes that is independent of physical inactivity.^15–17^ Research in nonpregnant populations suggests that higher levels of sedentary behavior are associated with a higher depression risk.^18,19^ Regarding sleep, sedentary behavior is associated with several poor sleep parameters.^20–23^ Fewer data on the potential risks of excessive sedentary behavior are available in pregnant populations, especially for sleep outcomes, but a recent systematic review suggests that longer sedentary time is associated with a higher risk of postpartum depression.^24^

Sedentary behavior reduction may be a promising, approachable behavioral strategy to improve psychological and sleep outcomes in pregnant populations who may struggle to add more intense physical activity as recommended. However, the current evidence base is largely observational. Therefore, the experimental evidence testing the effects of reducing sedentary behavior during pregnancy on changes in psychological and sleep outcomes should be explored.

The Sedentary Behavior Reduction in Pregnancy Intervention (SPRING) was a randomized multi-component pilot and feasibility intervention trial (*n* = 51) in the second and third trimesters of pregnancy that sought to reduce sedentary behavior while increasing standing and steps.^25,26^ The behavioral intervention included individualized coaching, a sit-stand desk, a wearable activity device, and a social media group, while the control group received a handout with physical activity recommendations. The intervention successfully reduced sedentary behavior by 0.84 hour/day and increased standing by 0.77 hour/day, with no significant changes in the control group. Psychological and sleep data were collected as exploratory outcomes and are the focus of this report. Here, we use data from the SPRING randomized clinical trial to:

1. Investigate the effect of the SPRING intervention on psychological and sleep outcomes across pregnancy compared with controls, and
2. Assess the relationship between changes in sedentary behavior and physical activity with changes in psychological and sleep outcomes during pregnancy, without regard to a randomized group.

## Methods

### Participants

Participants were enrolled in their first trimester of pregnancy and were at risk of both high sedentary behavior (to enable meaningful reductions with intervention) and adverse pregnancy outcomes (to intervene upon individuals at higher risk for these exploratory outcomes), as described previously.^25,26^ Eligible participants reported high sedentary behavior at screening, for example, a job that was primarily sitting or fewer than 6000 steps per day from a wearable activity monitor. In addition, participants had at least one risk factor for an adverse pregnancy outcome, including nulliparity, a body mass index (BMI) of at least 30 kg/m^2^, a history of an adverse outcome in a prior pregnancy, and/or being 35 years of age or older. Ethical approval was provided by the local review board, and all participants provided written informed consent.

### Assessments

Assessments were conducted in each trimester. Baseline assessments took place between 10^0^ and 12^6^ weeks of gestation, and second and third trimester follow-up assessments occurred between 20^0^−22^6^ and 32^0^−34^6^ weeks, respectively. Participants completed validated questionnaires to assess mood, quality of life, and sleep parameters.

Depressive symptoms were assessed using the Center for Epidemiological Studies—Depression scale (CES-D),^27^ in which scores ranged from 0 to 30 and a score ≥15 was indicative of elevated depressive symptoms. Perceived stress was ascertained from the Perceived Stress Scale (PSS),^28^ with scores ranging from 0 to 40. Higher PSS scores suggest greater perceived stress. Pregnancy quality of life was assessed using the Health-Related Quality of Life for Nausea and Vomiting during Pregnancy scale (NVPQoL),^29^ with higher scores suggesting worse quality of life. Overall mood was assessed using the 40-item profile of mood states.^4,30^ Total mood disturbance (TMD) was calculated by summing item scores for the tension, anger, fatigue, depression, esteem-related affect, and confusion subscales, then subtracting the summed item scores for the vigor subscale. A higher score indicates worse mood disturbance.

Sleep parameters were self-reported using the Pittsburgh Sleep Quality Index^31^ (PSQI). The instrument includes subscales for duration, sleep disturbances, sleep latency, daytime dysfunction, sleep efficiency, sleep quality, and medication use. The global PSQI score is calculated by summing each subscale; a score greater than 5 indicates poor sleep quality. In addition, factor scores were calculated for quality (the sum of the sleep quality, sleep latency, and medication use subscales), daily disturbances (the sum of the sleep disturbances and daytime dysfunction subscales), and efficiency (the sum of the sleep duration and sleep efficiency subscales).^32^ Finally, continuous values of sleep duration (hours) and sleep efficiency (percentage) were analyzed.

To assess sedentary behavior and physical activity, participants wore a thigh-mounted activPAL3 micro accelerometer (PAL Technologies, Glasgow, Scotland) continuously for one week in each trimester, removing it only for swimming activities, and completed a concurrent wear diary. After processing using PALTechnologies software, event files and daily summaries were downloaded, and a diary-informed cleaning approach was used to determine waking wear time. For those with at least 5 valid days of wear, sedentary behavior was analyzed both as total daily duration and percentage of total wear time. Time spent in moderate-to-vigorous physical activity (MVPA) was estimated as minutes per day during which the participant achieved at least 100 steps per minute from the daily summaries from valid days. Given that the intervention targeted sedentary behavior, we chose to only analyze device-based data because prior literature has shown that self-report measures such as the Pregnancy Physical Activity Questionnaire have low validity for sedentary behavior.^33^

### Randomization and intervention

The randomization and intervention protocol has been described in detail previously.^25,26^ In brief, participants were randomly allocated 2:1 to either a sedentary behavior reduction intervention or a no-contact control group following the baseline assessment. Intervention participants received a multi-level intervention that included biweekly individual behavioral coaching, inclusion in a study-administered social media group, a height-adjustable workstation, and a wearable fitness tracker. Behavioral sessions focused on decreasing sitting time by increasing standing and stepping behaviors. No-contact control participants were urged to maintain their usual activity patterns.

### Statistical analyses

Mixed-effect linear regression models using an interaction term between intervention group and trimester were used to examine whether the sedentary behavior reduction intervention yielded any changes in sleep, depressive symptoms, perceived stress, health-related quality of life, or mood parameters over the intervention period compared with controls. In addition, Spearman rank correlations between changes in behaviors (sedentary behavior, steps per day, and MVPA) with changes in mood, sleep, and quality of life outcomes from the baseline visit to the follow-up visits were examined in the full sample, without regard to the intervention group. Analyses were conducted using Stata version 18 (StataCorp, College Station, TX), and significance was accepted at *p* < 0.05.

## Results

Participants in the SPRING Study (*N* = 51) were on average (standard deviation [SD]) 32.0 (4.3) years old with a pre-pregnancy BMI of 28.0 (8.8) kg/m^2^. As shown in Table 1, no significant differences were found between the intervention (*n* = 34) and control (*n* = 17) groups for demographic or pregnancy characteristics, and the groups had similar baseline values for all mood, quality of life, and sleep parameters.

**Table 1.** Descriptive Characteristics and Baseline Scores for Psychological and Sleep Parameters by Randomized Group

	Intervention (*n* = 34)	Control (*n* = 17)	*p*-Value
Demographics			
Age, years	31.7 (4.7)	32.5 (3.6)	0.559
Race			0.075
White	25 (74)	17 (100)	
Black	6 (18)	0 (0)	
Other	3 (9)	0 (0)	
Ethnicity			0.610
Non-Hispanic	33 (97)	16 (94)	
Hispanic	1 (3)	1 (6)	
Employment			0.481
Full-time	22 (65)	13 (76)	
Part-time	3 (9)	2 (12)	
Not currently employed	9 (27)	2 (12)	
Pregnancy characteristics and history			
Gestational age at baseline, weeks	11.8 (0.8)	11.8 (0.6)	0.881
Pre-pregnancy BMI, kg/m^2^	28.1 (9.7)	28.1 (7.7)	0.978
Parity			0.480
Nulliparous	20 (59)	7 (41)	
1	9 (26)	6 (35)	
2 or more	5 (15)	4 (24)	
History of APO (among previously pregnant)			0.770
No previous history	5 (36)	3 (30)	
Yes previous APO	9 (64)	7 (70)	
Baseline outcome values			
Depressive symptoms	8.6 (4.7)	7.6 (3.2)	0.430
Perceived stress	15.3 (5.5)	13.6 (4.6)	0.291
Total mood disturbance	6.9 (12.2)	7.6 (11.1)	0.837
Nausea and vomiting in pregnancy quality of life	113.5 (32.4)	106.6 (35.8)	0.505
Global PSQI score	7.5 (3.2)	7.1 (3.9)	0.649
Quality factor score	3.5 (1.7)	2.9 (2.1)	0.308
Disturbances factor score	2.9 (1.0)	2.7 (0.9)	0.524
Efficiency factor score	1.2 (1.6)	1.5 (1.9)	0.583
Sleep duration (hours)	7.5 (1.4)	7.2 (1.3)	0.539
Percent efficiency	82.7 (13.8)	80.6 (10.7)	0.605

Data presented as mean (SD) or frequency (%). APO, adverse pregnancy outcome; BMI, body mass index; PSQI, Pittsburgh Sleep Quality Index.

### Effect of the intervention

**FIG. 1.**
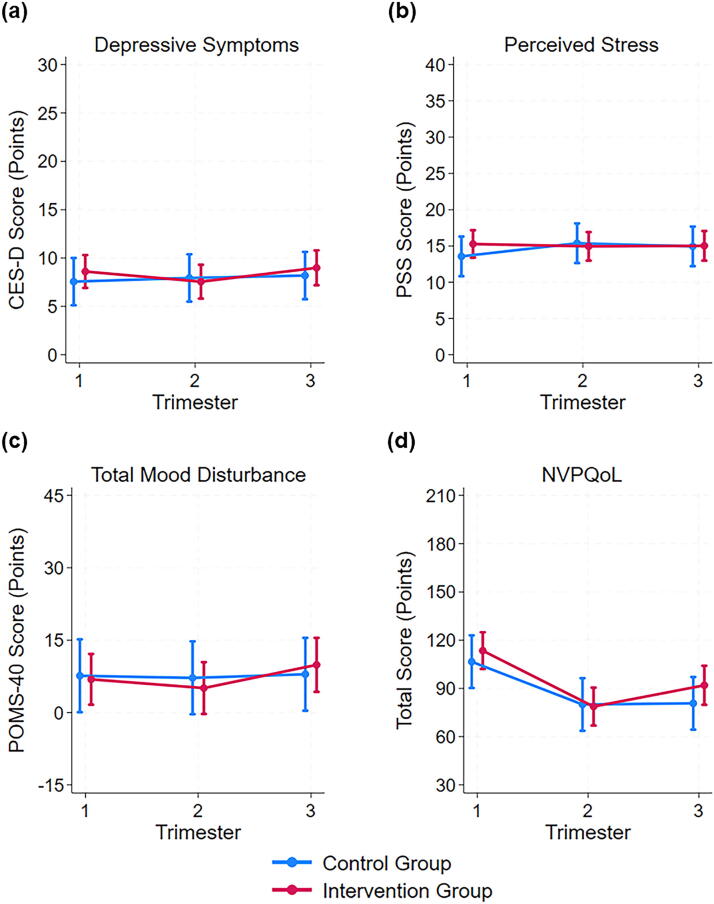
Psychological well-being and quality of life marginal mean scores (95% CI) at each trimester by intervention condition for **(a)** depressive symptoms, **(b)** perceived stress, **(c)** total mood disturbance, and **(d)** pregnancy nausea and vomiting quality of life; no significant time by intervention condition interactions were found (*p* > 0.05 for all). CES-D, centers for epidemiology depression scale; NVPQoL, Health-Related Quality of Life for Nausea and Vomiting during Pregnancy scale; POMS-40, profile of mood states 40-item scale; PSS, perceived Stress Scale.

Raw scores for outcome variables by trimester and group can be found in Supplementary Table S1. Results of linear mixed effects models found no significant interactions between intervention status and trimester for depressive symptoms, perceived stress, total mood disturbance, or NVPQoL (Fig. 1). No main effect of intervention status was found for any mood or quality of life outcome. The main effect of trimester was only significant for NVPQoL, in which nausea and vomiting quality of life symptoms improved in the second and third trimesters compared with the first (*p* = 0.001).

**FIG. 2.**
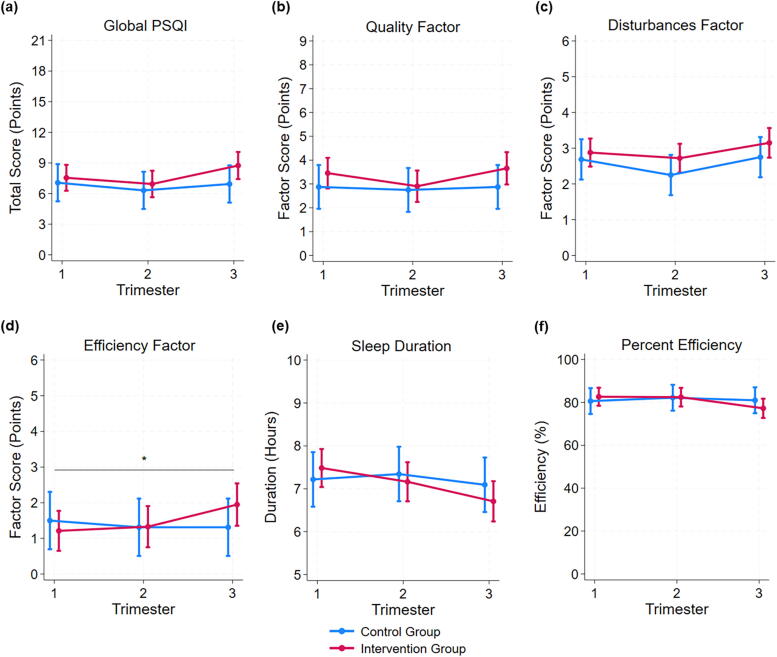
Sleep characteristic marginal means (95% CI) at each trimester by intervention condition for **(a)** the global PSQI score; factor scores for **(b)** quality, **(c)** disturbances, and **(d)** efficiency; **(e)** absolute sleep duration; and **(f)** absolute efficiency. *Significant interaction effect for time by intervention condition for the efficiency factor, *p* = 0.038. PSQI, Pittsburgh Sleep Quality Index.

Results of linear mixed effects models for the sleep parameters are shown in Figure 2. A significant interaction effect of the intervention group by trimester was found only for the efficiency factor score (Fig. 2d), in which those in the intervention group had worsening efficiency factor scores as pregnancy progressed while there was no change across pregnancy in the control group (*p* = 0.038). No significant main effects of either intervention status or trimester were found for any sleep parameters.

### Correlations with sedentary time and MVPA

As shown in Table 2, changes in sedentary time, steps per day, and MVPA from the first to second or the first to third trimesters were not correlated with changes in depressive symptoms, perceived stress, or total mood disturbance over the same time periods. Small-to-moderate significant correlations, ranging from |0.32| to |0.45|, were seen between changes in sedentary time, steps per day, and MVPA and changes in NVPQoL symptoms. Less sedentary time, more steps per day, and greater MVPA were each correlated with decreased NVPQoL symptoms, and these correlations were stronger when comparing the change from the first to the third trimesters versus the change from the first to the second trimesters.

**Table 2.** Spearman Rank Correlations Between Changes in Mood/Quality of Life Parameters and Changes in Movement Behaviors from the First to Second and First to Third Trimesters

	CES-D	PSS	TMD	NVPQoL
Change from first to second trimester				
Sed time (hours)	0.12	0.13	0.07	0.18
Sed time (percent of wear time)	0.05	0.01	0.12	0.40^*^
Steps per day	0.12	0.02	−0.09	−0.32^*^
MVPA	0.02	−0.12	−0.26	−0.34^*^
Change from first to third trimester				
Sed time (hours)	0.15	0.05	0.10	0.34^*^
Sed time (percent of wear time)	0.06	0.001	0.02	0.45^*^
Steps per day	0.08	0.16	0.01	−0.40^*^
MVPA	0.06	0.07	−0.08	−0.36^*^

A positive change means the later trimester value is higher than the first trimester value.

^*^ Indicates *p* < 0.05.

CES-D, Center for Epidemiological Studies—Depression scale; MVPA, moderate-to-vigorous physical activity; NVPQoL, Health-Related Quality of Life for Nausea and Vomiting during Pregnancy scale; PSS, Perceived Stress Scale; Sed, sedentary; TMD, total mood disturbance.

Correlations of changes in sedentary time, steps per day, and MVPA with changes in sleep parameters are shown in Table 3. When looking at the changes from the first to the second trimester, small but significant positive correlations were found between increases in sedentary time and unfavorable increases in the global PSQI score, increases in steps per day with favorable increases in sleep duration and sleep efficiency, and increases in MVPA with favorable increases in sleep duration. A small, significant negative correlation was found between increases in MVPA and favorable decreases in the efficiency factor score. When looking at changes from the first to the third trimesters, only a small significant negative correlation was found between increases in MVPA and favorable decreases in the quality factor score.

**Table 3.** Spearman Rank Correlations Between Changes in Sleep Parameters and Changes in Movement Behaviors from the First to Second and First to Third Trimesters

	Global PSQI	Quality factor	Disturbances factor	Efficiency factor	Sleep duration	Percent efficiency
Change from first to second trimester						
Sed time (hours)	0.16	0.18	0.08	−0.07	−0.12	0.05
Sed time (percent of wear time)	0.30^*^	0.24	0.05	0.13	−0.24	−0.21
Steps per day	−0.15	−0.05	−0.02	−0.29	0.31^*^	0.34^*^
MVPA (minutes per week)	−0.09	0.07	−0.03	−0.32^*^	0.31^*^	0.28
Change from first to third trimester						
Sed time (hours)	0.22	0.16	0.14	0.12	−0.11	−0.09
Sed time (percent of wear time)	0.15	0.17	0.19	0.02	0.11	−0.003
Steps per day	−0.12	−0.24	−0.002	0.03	−0.05	0.04
MVPA (minutes per day)	−0.25	−0.33^*^	0.08	−0.16	0.16	0.11

A positive change means the later trimester value is higher than the first trimester value.

^*^ Indicates *p* < 0.05.

MVPA, moderate-to-vigorous physical activity; PSQI, Pittsburgh Sleep Quality Index; Sed, sedentary.

## Discussion

Our study found that the SPRING intervention had no effect on measures of psychological factors during pregnancy; however, surprisingly, intervention participants demonstrated a significant worsening of the sleep efficiency factor score over pregnancy compared with control participants. Yet, more consistent with our expectations, we found several small significant correlations between increases in physical activity and decreases in sedentary behavior with beneficial changes in nausea/vomiting quality of life scores and several sleep parameters. These findings suggest a possible relationship between changes in physical activity and sedentary behavior with changes in nausea/vomiting quality of life and sleep health which could be bidirectional or in the reverse direction—where beneficial changes in the outcomes as pregnancy progresses lead to less sedentary behavior and more physical activity.

Our findings that the intervention did not improve psychological outcomes across pregnancy align with other studies, including interventions, in nonpregnant adults that found no association between sedentary behavior reduction and psychological factors.^34^ Changes in mentally active rather than mentally passive sedentary behavior may explain why there were no differences in psychological factors between groups in the SPRING intervention.^35^ A meta-analysis found that mentally passive sedentary behavior, such as television watching, is associated with increased depression risk, while mentally active sedentary behavior, which includes working on a computer, is not.^36^ Since the SPRING intervention participants were provided with a sit-stand desk, it is likely that they mainly decreased their mentally active sedentary behavior. If more leisure or mentally passive sedentary behaviors were decreased, there may have been a significant intervention effect on depressive symptoms and possibly other psychological factors. Furthermore, average depressive symptoms, perceived stress, and mood disturbances were low across pregnancy in both groups, which could have resulted in a floor effect. We may have observed an intervention benefit in a sample with a poorer psychological profile at baseline.

It is unclear why the intervention group experienced significantly worsening sleep efficiency factor scores compared with the control group. Observational evidence in pregnant women suggests that those with low sleep efficiency may engage in less sedentary behavior, and those with longer sleep duration also accumulate more sedentary time,^37,38^ and our findings indicate that reducing sedentary behavior may cause lower sleep efficiency and duration. Baseline demographics, pregnancy characteristics, psychological factors, and sleep parameters were similar across groups, so it is unlikely issues such as parity were confounding factors. Additionally, the main findings of SPRING reported that 54% of intervention participants reported being more comfortable, 42% reported less pain, and 42% reported less swelling after completing the intervention.^25^ Therefore, it is unlikely that worsening sleep efficiency and duration were due to increased musculoskeletal pain from the intervention effect of increased standing. Future research is needed to understand the mechanisms that could explain this unexpected finding. Of note, the mean global PSQI score ranged between 6.3 and 8.9 across trimesters for both groups at all three time points (see Supplementary Table S1). This finding aligns with previous studies that have found that sleep is generally poor during pregnancy.^5,39^

When we examined correlations between changes in movement behaviors and changes in psychological and sleep outcomes without regard to the intervention group, we found that decreases in sedentary behavior and increases in physical activity between trimesters were only correlated with beneficial changes in the nausea/vomiting quality of life scores and sleep parameters. In line with these findings, a recent study of pregnant women found that moderate-intensity physical activity was associated with better quality of life.^40^ Given that we did not find a significant effect of the intervention, one plausible explanation is that those with declining symptoms of nausea or vomiting were able to participate in a more active, less sedentary lifestyle (rather than more activity causing an easing of symptoms). This is consistent with reported facilitators of sedentary behavior and barriers to physical activity in pregnancy that include pregnancy-related symptoms of nausea and fatigue.^14,41^ Regarding sleep parameters, we found a general trend that more physical activity was associated with better sleep efficiency, duration, and quality; sedentary time was not correlated with sleep parameters in this sample. These findings are in line with previous literature in pregnant and nonpregnant adults. A longitudinal study of over 2000 pregnant women found that physical activity was positively associated with healthy sleep parameters,^42^ and a systematic review and meta-analysis in nonpregnant adults found that prolonged sedentary behavior was associated with less healthy sleep parameters such as increased risk of insomnia, more sleep disturbances, and poorer sleep quality.^20^ Another study aligning with our findings was a pregnancy cohort study comparing sedentary behavior and sleep duration in individuals who were pregnant prior to versus during the COVID pandemic.^38^ This study found that the pre-COVID participants had less sedentary behavior and shorter sleep during the third trimester than the pandemic-era participants.

Although our study uncovered the effects of the SPRING intervention on psychological and sleep parameters along with correlations between specific behavior and outcome changes, it has limitations. Being a pilot study, the sample size of SPRING was small. Also, the randomization of SPRING was 2:1 in favor of the intervention group, which further limited our power to detect between-group differences. Finally, device-based sleep data were not obtained; self-reported and device-based data are only mildly correlated with each other and often provide complementary insights about separate sleep domains.^43^ Considering these limitations, the results of this study should be verified in a larger sample and include a multi-modal assessment of sleep.

Despite these limitations, this study had several strengths. First, SPRING employed a randomized controlled trial study design, a rigorous approach to investigating the causal relationships between the intervention (which successfully reduced sedentary behavior) and outcomes. Next, SPRING tracked participants during each pregnancy trimester, providing a better understanding of behavior and outcome changes across pregnancy. SPRING also used a best practice, device-based sedentary behavior measurement *via* thigh-worn accelerometry, which uniquely measures posture and intensity and has been validated for pregnant populations.^33^

## Conclusion

Contrary to our hypothesis, the SPRING intervention did not improve depressive symptoms, stress, pregnancy quality of life, and overall mood but had a negative effect on self-reported sleep efficiency factor scores during the third trimester of pregnancy. Increases in physical activity were more often positively correlated with quality of life as related to nausea and vomiting and sleep parameters than sedentary behavior. Therefore, although decreasing sedentary behavior may be a more feasible or approachable behavior change during pregnancy than increasing physical activity, it may be less effective for improving psychological and sleep health. Future research should verify our findings with a larger sample size, and interventions aiming to decrease sedentary behavior in pregnant women may also target decreases in mentally passive sedentary behavior (*e.g.,* leisure screen time) instead of mentally active sedentary behavior.

## Abbreviations Used

APO: adverse pregnancy outcome
BMI: body mass index
CES-D: Center for Epidemiological Studies—Depression scale
MVPA: moderate-to-vigorous physical activity
NVPQoL: Health-Related Quality of Life for Nausea and Vomiting during Pregnancy scale
POMS-40: Profile of Mood States 40-item scale
PSQI: Pittsburgh Sleep Quality Index
PSS: Perceived Stress Scale
SPRING: Sedentary Behavior Reduction in Pregnancy Intervention
TMD: total mood disturbance

## References

[1] Van Niel MS, Payne JL. Perinatal depression: A review. Cleve Clin J Med 2020;87(5):273–277; doi: 10.3949/ccjm.87a.19054 32357982

[2] Eick SM, Barrett ES, van ‘t Erve TJ, et al. Association between prenatal psychological stress and oxidative stress during pregnancy. Paediatr Perinat Epidemiol 2018;32(4):318–326; doi: 10.1111/ppe.12465 29603338 PMC6103836

[3] Deo BK, Sapkota N, Kumar R, et al. A study on pregnancy, perceived stress and depression. JBP Koirala Inst Health Sci 2020;3(1):79–87; doi: 10.3126/jbpkihs.v3i1.30331

[4] Li H, Bowen A, Bowen R, et al. Mood instability during pregnancy and postpartum: A systematic review. Arch Womens Ment Health 2020;23(1):29–41; doi: 10.1007/s00737-019-00956-6 30834475

[5] Sedov ID, Cameron EE, Madigan S, et al. Sleep quality during pregnancy: A meta-analysis. Sleep Med Rev 2018;38:168–176; doi: 10.1016/j.smrv.2017.06.005 28866020

[6] Staneva A, Bogossian F, Pritchard M, et al. The effects of maternal depression, anxiety, and perceived stress during pregnancy on preterm birth: A systematic review. Women Birth 2015;28(3):179–193; doi: 10.1016/j.wombi.2015.02.003 25765470

[7] Facco FL, Parker CB, Hunter S, et al.; NICHD NuMoM2b and NHLBI NuMoM2b Heart Health Study Networks. Association of adverse pregnancy outcomes with self-reported measures of sleep duration and timing in women who are nulliparous. J Clin Sleep Med 2018;14(12):2047–2056; doi: 10.5664/jcsm.7534 30518449 PMC6287730

[8] Sharma SK, Nehra A, Sinha S, et al. Sleep disorders in pregnancy and their association with pregnancy outcomes: A prospective observational study. Sleep Breath 2016;20(1):87–93; doi: 10.1007/s11325-015-1188-9 25957617

[9] Kandola A, Ashdown-Franks G, Hendrikse J, et al. Physical activity and depression: Towards understanding the antidepressant mechanisms of physical activity. Neurosci Biobehav Rev 2019;107:525–539; doi: 10.1016/j.neubiorev.2019.09.040 31586447

[10] Costa DD, Rippen N, Dritsa M, et al. Self-reported leisure-time physical activity during pregnancy and relationship to psychological well-being. J Psychosom Obstet Gynaecol 2003;24(2):111–119; doi: 10.3109/01674820309042808 12854395

[11] Baker JH, Rothenberger SD, Kline CE, et al. Exercise during early pregnancy is associated with greater sleep continuity. Behav Sleep Med 2018;16(5):482–493; doi: 10.1080/15402002.2016.1228649 27739877 PMC6124311

[12] American College of Obstetrics and Gynecology. Exercise During Pregnancy. 2024. Available from: https://www.acog.org/womens-health/faqs/exercise-during-pregnancy [Last accessed: August 11, 2024].

[13] Hesketh KR, Evenson KR, Stroo M, et al. Physical activity and sedentary behavior during pregnancy and postpartum, measured using hip and wrist-worn accelerometers. Prev Med Rep 2018;10:337–345; doi: 10.1016/j.pmedr.2018.04.012 29868389 PMC5984239

[14] Harrison AL, Taylor NF, Shields N, et al. Attitudes, barriers and enablers to physical activity in pregnant women: A systematic review. J Physiother 2018;64(1):24–32; doi: 10.1016/j.jphys.2017.11.012 29289592

[15] Paley JL, Jones MA, Catov JM, et al. Associations of physical activity and sedentary behaviors with depressive symptoms and mood disturbance throughout pregnancy. J Womens Health (Larchmt) 2024;33(8):1128–1138; doi: 10.1089/jwh.2023.0419 38324012 PMC11392679

[16] Barone Gibbs B, Jones MA, Jakicic JM, et al. Objectively measured sedentary behavior and physical activity across 3 trimesters of pregnancy: The monitoring movement and health study. J Phys Act Health 2021;18(3):254–261; doi: 10.1123/jpah.2020-0398 33508775 PMC8054065

[17] Jones MA, Catov JM, Jeyabalan A, et al. Sedentary behaviour and physical activity across pregnancy and birth outcomes. Paediatr Perinat Epidemiol 2021;35(3):341–349; doi: 10.1111/ppe.12731 33124060 PMC8186559

[18] Teychenne M, Ball K, Salmon J. Sedentary behavior and depression among adults: A review. Int J Behav Med 2010;17(4):246–254; doi: 10.1007/s12529-010-9075-z 20174982

[19] Zhai L, Zhang Y, Zhang D. Sedentary behaviour and the risk of depression: A meta-analysis. Br J Sports Med 2015;49(11):705–709; doi: 10.1136/bjsports-2014-093613 25183627

[20] Yang Y, Shin JC, Li D, et al. Sedentary behavior and sleep problems: A systematic review and meta-analysis. Int J Behav Med 2017;24(4):481–492; doi: 10.1007/s12529-016-9609-0 27830446

[21] Koohsari MJ, Yasunaga A, McCormack GR, et al. Sedentary behaviour and sleep quality. Sci Rep 2023;13(1):1180; doi: 10.1038/s41598-023-27882-z 36670182 PMC9859796

[22] Jeong SH, Jang BN, Kim SH, et al. Association between sedentary time and sleep quality based on the Pittsburgh Sleep Quality Index among South Korean adults. BMC Public Health 2021;21(1):2290; doi: 10.1186/s12889-021-12388-y 34911512 PMC8675446

[23] Hargens TA, Scott MC, Olijar V, et al. Markers of poor sleep quality increase sedentary behavior in college students as derived from accelerometry. Sleep Breath 2021;25(1):537–544; doi: 10.1007/s11325-020-02190-2 32948936

[24] Osumi A, Kanejima Y, Ishihara K, et al. Effects of sedentary behavior on the complications experienced by pregnant women: A systematic review. Reprod Sci 2024;31(2):352–365; doi: 10.1007/s43032-023-01321-w 37644379

[25] Gibbs BB, Kozai AC, McAdoo SN, et al. The sedentary behavior reduction in pregnancy intervention (SPRING) pilot and feasibility randomized trial. BMC Pregnancy Childbirth 2024;24(1):261; doi: 10.1186/s12884-024-06474-3 38605328 PMC11007988

[26] Barone Gibbs B, Kozai AC, McAdoo SN, et al. Rationale, design, and methods for the Sedentary Behavior Reduction in Pregnancy Intervention (SPRING): Protocol for a pilot and feasibility randomized controlled trial. JMIR Res Protoc 2023;12(1):e48228; doi: 10.2196/48228 37314845 PMC10337422

[27] Eaton WW, Muntaner C, Smith C, et al. Center for Epidemiology Studies Depression Scale: review and revision (CESD and CESD-R). In: The Use of Psychological Testing for Treatment Planning and Outcomes assessment: INstrucment for Adults, Vol 3, 3rd ed. Lawrence Erlbaum Associates Publishers; Mahwwah, NJ, USA; 2004, pp. 363–377.

[28] Cohen S. Measuring Stress: A Guide for Health and Social Scientists. Oxford University Press; 1997.

[29] Munch S, Korst LM, Hernandez GD, et al. Health-related quality of life in women with nausea and vomiting of pregnancy: The importance of psychosocial context. J Perinatol 2011;31(1):10–20; doi: 10.1038/jp.2010.54 20410906 PMC3511856

[30] Grove J, Prapavessis H. Preliminary evidence for the reliability and validity of an abbreviated Profile of Mood States. Int J Sport Psychol 1992;23(2):93–109.

[31] Buysse DJ, Reynolds CF, 3rd, Monk TH, et al. The Pittsburgh Sleep Quality Index: A new instrument for psychiatric practice and research. Psychiatry Res 1989;28(2):193–213; doi: 10.1016/0165-1781(89)90047-4 2748771

[32] Cole JC, Motivala SJ, Buysse DJ, et al. Validation of a 3-factor scoring model for the Pittsburgh sleep quality index in older adults. Sleep 2006;29(1):112–116; doi: 10.1093/sleep/29.1.112 16453989

[33] Barone Gibbs B, Paley JL, Jones MA, et al. Validity of self-reported and objectively measured sedentary behavior in pregnancy. BMC Pregnancy Childbirth 2020;20(1):99; doi: 10.1186/s12884-020-2771-z 32046663 PMC7014698

[34] Teychenne M, Stephens LD, Costigan SA, et al. The association between sedentary behaviour and indicators of stress: A systematic review. BMC Public Health 2019;19(1):1357; doi: 10.1186/s12889-019-7717-x 31647002 PMC6813058

[35] Kikuchi H, Inoue S, Sugiyama T, et al. Distinct associations of different sedentary behaviors with health-related attributes among older adults. Prev Med 2014;67:335–339; doi: 10.1016/j.ypmed.2014.08.011 25117527

[36] Huang Y, Li L, Gan Y, et al. Sedentary behaviors and risk of depression: A meta-analysis of prospective studies. Transl Psychiatry 2020;10(1):26; doi: 10.1038/s41398-020-0715-z 32066686 PMC7026102

[37] Whitaker KM, Zhang D, Kline CE, et al. Associations of sleep with sedentary behavior and physical activity patterns across pregnancy trimesters. Womens Health Issues 2021;31(4):366–375; doi: 10.1016/j.whi.2021.02.003 33715925 PMC8428394

[38] Kozai AC, Jones MA, Borrowman JD, et al. Patterns of physical activity, sedentary behavior, and sleep across pregnancy before and during two COVID pandemic years. Midwifery 2025;141:104268; doi: 10.1016/j.midw.2024.104268 39721225 PMC11758526

[39] Yang Y, Li W, Ma T-J, et al. Prevalence of poor sleep quality in perinatal and postnatal women: A comprehensive meta-analysis of observational studies. Front Psychiatry 2020;11:161; doi: 10.3389/fpsyt.2020.00161 32231599 PMC7082815

[40] Song B, Wang D, Yan X, et al. Physical activity and sleep quality among pregnant women during the first and second trimesters are associated with mental health and adverse pregnancy outcomes. BMC Womens Health 2024;24(1):455; doi: 10.1186/s12905-024-03126-8 39138442 PMC11321155

[41] Gibbs BB, Jones MA, Whitaker KM, et al. Measurement of barriers, attitudes, and expectations for sitting less in pregnancy. Am J Health Behav 2021;45(6):956–970; doi: 10.5993/AJHB.45.6.1 34969408 PMC9243676

[42] Tan L, Zou J, Zhang Y, et al. A longitudinal study of physical activity to improve sleep quality during pregnancy. Nat Sci Sleep 2020;12:431–442; doi: 10.2147/NSS.S253213 32765140 PMC7367923

[43] Okun ML, Kohl V, Feliciano L. Comparison of longitudinal diary and actigraphy-assessed sleep in pregnant women. Sleep Med 2021;88:149–156; doi: 10.1016/j.sleep.2021.09.015 34753041

