# Supplementary material for: Influence of a Sedentary Behavior Intervention on Mood, Sleep, and Quality of Life Outcomes During Pregnancy: The SPRING Study
Source: Womens Health Rep (New Rochelle). 2025 Mar 25;6(1):305–14. doi: 10.1089/whr.2024.0176 (PMC12040537; doi:10.1089/whr.2024.0176)
Supplement: Supplementary Table S1 [file whr.2024.0176_supplementary_table_s1.pdf]

Table S1. Mean (SD) scores for mood, quality of life, and sleep parameters for each group at each visit.

	Trimester 1	Trimester 2	Trimester 3
**Depressive symptoms**			
Intervention	8.6 (4.7)	7.7 (5.8)	8.9 (5.8)
Control	7.6 (3.2)	7.9 (4.2)	8.2 (5.5)
**Perceived stress**			
Intervention	15.3 (5.5)	14.6 (5.8)	14.8 (6.3)
Control	13.6 (4.6)	15.4 (4.7)	14.9 (6.3)
**Total mood disturbance**			
Intervention	6.9 (12.2)	5.5 (17.6)	10.1 (18.6)
Control	7.6 (11.1)	7.2 (17.9)	7.9 (14.7)
**Nausea and vomiting quality of life**			
Intervention	113.5 (32.4)	80.7 (37.7)	90.7 (39.6)
Control	106.6 (35.8)	80.0 (23.3)	80.8 (22.7)
**Pittsburgh Sleep Quality Index global score**			
Intervention	7.5 (3.2)	7.1 (3.8)	8.9 (4.5)
Control	7.1 (3.9)	6.3 (3.8)	6.9 (3.7)
**Quality Factor**			
Intervention	3.5 (1.7)	3.0 (1.9)	3.8 (2.3)
Control	2.9 (2.1)	2.8 (2.0)	2.9 (1.4)
**Disturbances Factor**			
Intervention	2.9 (1.0)	2.8 (1.3)	3.1 (1.2)
Control	2.7 (0.9)	2.3 (1.2)	2.8 (1.2)
**Efficiency Factor**			
Intervention	1.2 (1.6)	1.3 (1.5)	1.9 (1.8)
Control	1.5 (1.9)	1.3 (1.6)	1.3 (1.8)
**Sleep Duration (h)**			
Intervention	7.5 (1.4)	7.2 (1.2)	6.8 (1.2)
Control	7.2 (1.3)	7.3 (1.4)	7.1 (1.6)
**Efficiency (%)**			
Intervention	82.7 (13.8)	82.4 (11.1)	76.5 (12.5)
Control	80.6 (10.7)	82.2 (13.0)	81.0 (14.2)
